# Editorial: Hormones, Neurotransmitters, and T-Cell Development in Health and Disease

**DOI:** 10.3389/fendo.2019.00454

**Published:** 2019-07-11

**Authors:** Ana Rosa Pérez, Daniella Areas Mendes-da-Cruz, Vincent Geenen, Wilson Savino

**Affiliations:** ^1^Institute of Clinical and Experimental Immunology of Rosario, (IDICER-National Scientific and Technical Research Council), Rosario, Argentina; ^2^Laboratory on Thymus Research, Oswaldo Cruz Institute, Oswaldo Cruz Foundation, Rio de Janeiro, Brazil; ^3^National Institute of Science and Technology on Neuroimmunomodulation, Oswaldo Cruz Institute, Oswaldo Cruz Foundation, Rio de Janeiro, Brazil; ^4^Department of Biomedical and Preclinical Sciences, GIGA-I3 Center of Immunoendocrinology, GIGA Research Institute, University of Liège, Liège, Belgium

**Keywords:** thymus, hormones, neurottransmitters, T lymhocytes, thymopoiesis

Thymus physiology, T-cell development, and peripheral T-cell homeostasis are controlled by a large variety of soluble molecules and their cognate receptors, targeting both the lymphoid and non-lymphoid compartments. Hormones, neurotransmitters, and cytokines influence the functions of distinct microenvironmental cells, including their maturation, survival, and antigen presentation. Additionally, they affect thymocyte survival, migration and selection, thus shaping the pool of mature T-cells in the periphery. Importantly, some of these circuits can be affected in pathological states.

Beneficial effects of the somatotrope axis on thymopoiesis have been extensively reported. Most of the data derive from studies carried out in mouse models with multiple pituitary deficiencies (i.e., lacking GH, PRL, and thyrotropic hormones), making it difficult to identify the real effect of GH on T cell homeostasis. Here, Bodart et al. show a series of studies carried out in GhrhKO mice, revealing the absence of thymic involution (in terms of relative weight or cellularity), accompanied only by minor changes in the proportions of thymocyte subsets. Authors also obtained data compatible with a faster commitment of double negative thymocytes in the thymopoietic process accompanied by an increased thymic output of naïve T cells, this later observation being consistent with a reduction of central memory T cells in secondary lymph organs. Taken together, these findings point out that the integrity of the GHRH/GH/IGF-1 axis is not required for thymocyte and peripheral T cell homeostasis in basal conditions, although it can influence the splenic B cell compartment. Overall, these data suggest that GH beneficial effects upon thymus homeostasis may be rather related with the positive counter regulatory effects of GH and PRL against the stress caused by glucocorticoids.

Additionally, it is known that with aging, thymic involution is accompanied by a diminution the thymopoietic capacity. The thymus is highly innervated by noradrenergic fibers and there is also a local production of norepinephrine, and both thymocytes and microenvironmental cells express adrenergic receptors. Leposavić and Pilipović examined the influence of sex steroids upon thymopoiesis during both perinatal/peripubertal evolving periods. The authors show that such hormones alter the adrenergic thymic microenvironment, although the mechanisms are still unknown. Yet, the available data insinuate that pharmacological handling of noradrenergic effects upon the thymus may improve the deleterious effects of aging on thymopoiesis.

Glucocorticoids can act on practically all types of cells, particularly T cells, showing to have upon these cells important immunosuppressive and anti-inflammatory effects, but also affect their phenotype, secretion profile, as well as survival and migratory properties. Here, Liberman et al. explored deeply the complexity of glucocorticoid effects upon immune cells (particularly on developing T cells), and the glucocorticoid receptor-mediated regulation. Importantly, authors also discuss how glucocorticoids induce both paradoxical anti- and pro-inflammatory responses, particularly in certain areas of the brain, in order to develop more effective treatments and avoid side effects, like toxicity and drug resistance.

Thymocyte-microenvironmental cell interactions are known to be critical for thymus homeostasis. The studies made by Oliveira-de-Abreu et al. strengthens the notion that changes in thymocyte/microenvironmental cell contact cause profound thymic alterations. For example, in the absence of Galectin-3 (Gal-3; a molecule abundantly expressed in the thymus that acts as a de-adhesive factor), the thymus showed abnormalities in developing T-cell number, proliferation, and death, with proportional enlargement of the DN1 compartment and a severe disorganization of the epithelial network. These findings reveal that Gal-3 is not only relevant for thymocyte homeostasis, but also to the maintenance of thymic architecture. Strikingly, glucocorticoid secretion is enhanced in the absence of Gal-3, possibly by a rise of both the adrenal and thymic steroidogenic machinery. How Gal-3 regulates glucocorticoid secretion has not yet been established and demands further studies. In contrast, Muñoz et al. adduce that not necessarily a disorder of the thymic epithelial network results in altered T development. Previous studies of the group showed that Ephrins (Eph) and their receptors seem to be relevant in both temporal and topologic thymocyte/thymic epithelial cell (TEC) interactions, modulating intrathymic thymocyte migration. Mice deficient in EphB2 and EphB3 showed severe abnormalities in TEC morphology, but relatively normal thymocyte subpopulations and immunocompetence. Here, the authors provide evidence supporting the notion that, regardless of Eph-deficient mice exhibited an altered epithelium, their TECs could express all necessary molecules to support thymocyte development and differentiation.

Two additional articles focused on diabetes, discussing possible relationships between thymic alterations, the development of autoreactive T lymphocytes and the establishment of abnormal immunoendocrine and metabolic profiles. Mendes-da-Cruz et al. discuss thymic disturbances observed in a well-established model of type-1 diabetes, as are NOD (non-obese diabetic) mice. A hallmark of the NOD thymus is the presence of enriched areas in simple-positive CD4^+^ and CD8^+^ thymocytes, possibly secondary to a defective expression of the fibronectin receptor VLA-5. It is well-known that diminished insulin-derived peptide expression in the thymus may favor the breakdown of insulin tolerance. Nevertheless, recent data show that expression of several miRNA is altered in the thymus of NOD mice, suggesting that some of them are involved in the mechanism underlying the generation of autoreactive cells. In addition, Andreone et al. make an insightful review of how hormones and neurotransmitters influence diverse T compartments under metabolic imbalance triggered by diabetes and their role in aggravating the disease.

Last, D'Attilio et al. and Pérez et al. review the immunoendocrine alterations reported during tuberculosis and Chagas disease, respectively. Although the real impact at the thymic level of both diseases in humans requires even more studies, data from experimental models strongly suggest that thymic abnormalities that take place in response to *Micobacterium tuberculosis* and *Trypanosoma cruzi* could act as contributing factors to pathology.

In the last years our understanding of the intricate network of neurotransmitters, hormones, cytokines and other molecules that play a major role in the regulation of T cell development and biological functions has increased enormously, but substantial challenges remain to provide a more comprehensive picture. The articles presented in this Research Topic reveal new evidence that illustrates the complex circuitries affecting the thymus and T cell homeostasis in both health and disease and point to further directions of future research.

## Author Contributions

AP wrote the manuscript with input from all authors, who made substantial contributions to the Editorial and approved it for publication.

### Conflict of Interest Statement

The authors declare that the research was conducted in the absence of any commercial or financial relationships that could be construed as a potential conflict of interest.

